# Efficacy and Molecular Mechanisms of Nystatin Against *Botrytis cinerea* on Postharvest Table Grape

**DOI:** 10.3390/foods13223624

**Published:** 2024-11-13

**Authors:** Yingying Wu, Shen Zhang, Jingyi Wang, Fan He, Haocheng Wei, Dongxiao Chen, Ying Wang

**Affiliations:** College of Ocean Food and Biological Engineering, Jimei University, Xiamen 361021, China

**Keywords:** gray mold, postharvest pathogenic fungi, antifungal mechanism, transcriptome, autophagy

## Abstract

The primary cause of postharvest loss in table grape fruit is attributed to gray mold, which is caused by *Botrytis cinerea*. The present study confirmed the inhibitory effects of nystatin on the growth and development of *B. cinerea*, which led to a remarkable reduction in the severity of gray mold on table grape fruits. Furthermore, the application of nystatin disrupted the membrane permeability of *B. cinerea*, causing increased cellular leakage and cell death. In addition, the transcriptome analysis showed that the application of nystatin effectively modulated the transcriptional profile of genes involved in ribosome and mitochondrion biogenesis, as well as oxidoreductase activity, thereby disrupting the homeostasis of cellular organelles. Moreover, the nystatin treatment down-regulated genes associated with membrane trafficking, protein degradation by the ubiquitin–proteasome system, and the autophagy process, ultimately attenuating the pathogenicity of *B. cinerea*. Collectively, nystatin can be considered a viable agent for managing gray mold on table grape fruit.

## 1. Introduction

Grape (*Vitis vinifera* L.) is globally cultivated for a variety of purposes such as the production of juices, wine, and raisins. The composition of table grapes includes a high content of carbohydrates, minerals, vitamins, and antioxidants [1], rendering them highly favored among consumers. However, the postharvest losses of table grapes are substantial, and their shelf life is limited due to their high sensitivity to the conditions during postharvest treatment, storage, transportation, and sales [2]. Fungal decay has been identified as a significant contributing factor leading to huge economic losses in the table grape marketing chain, resulting in an estimated loss of approximately 10–40% of the total grape production value [3,4]. Grapes possess a heightened susceptibility to *Botrytis cinerea*, a pathogen that frequently causes severe gray mold on grape berries [5,6]. The uncontrolled infection by *B. cinerea* can lead to the proliferation of airborne mycelium and rapid transmission to adjacent grape berries, resulting in a significant deterioration in quality [7]. It is a significant challenge to safely and effectively manage the gray mold of grapes. Fumigation with sulfur dioxide has long been extensively utilized for the preservation of grapes during storage [8]. However, the improper use of sulfur dioxide may result in bleaching of the fruit and browning of the rachis [9]. To date, a diverse range of alternative grape preservation techniques has been extensively reported [10,11]. However, the majority of the methods involve synthetic fungicides, which present hazards to both human health and the environment. Recently, as the concepts of ecological conservation, food safety, green production, and sustainable development have deeply resonated within society, the scrutiny of chemical pesticides has significantly intensified. Consequently, the development of biological pesticides characterized by high efficacy, low toxicity, minimal residue, and environmental friendliness has emerged as the main direction for future pesticide innovation. Within this realm, microbially derived agricultural antibiotics hold a vital position. *Streptomyces*, which resides in soil, possesses intricate secondary metabolic pathways that yield numerous biologically active molecules, accounting for approximately half of the microbially derived antibiotics utilized in both clinical and agricultural applications [12,13,14,15,16,17,18]. One prominent example is anisomycin, a pyrrolidine antibiotic that is a vital component of agricultural antibiotic 120. Widely applied in China, anisomycin effectively combats crop decay, safeguarding an estimated 13 million acres of farmland annually [15,16]. Additionally, salinomycin, renowned for its potent inhibition of pathogenic bacterial growth, has also gained extensive adoption in agriculture [17].

Recently, microbial secondary metabolites have attracted growing attention as potent active ingredients for the prevention and control of postharvest diseases caused by a range of plant pathogenic fungi; these include rapamycin [19,20], tanespimycin [19], natamycin [21], amphotericin B [22], and lucensomycin [23]. Nystatin is a polyene antibiotic derived from *Streptomyces noursei*, and exhibits efficacy against various fungal infections in humans [24]. It has been extensively utilized in the treatment of superficial fungal infections, as both oral and topical therapies [25]. As a non-absorbable antifungal medication, nystatin offers an effective prophylactic option owing to its ability to inhibit fungal colonization, excellent safety profile, and lack of drug–drug interactions. The prophylactic administration of nystatin may be considered for the management of children with congenital heart disease, cystic fibrosis, and chronic kidney disease [26]. Furthermore, the combination of nystatin–intralipid with caspofungin has been demonstrated to have enhanced antifungal activity against *Aspergillus terreus* compared to nystatin alone [27]. Kim et al. (2018) [28] developed a novel tissue conditioner containing alginate microspheres loaded with nystatin, which effectively regulated the release of this antifungal agent. The results from the antifungal assay demonstrated its potent efficacy against *Candida albicans*. The combination of nystatin and carvacrol has been demonstrated to cause a significant reduction in the minimum inhibitory concentration (MIC) against *Candida* species [29]. Moreover, the antifungal activity of nystatin against *Candida* biofilms is enhanced by alginate oligosaccharides, resulting in an up to a 32-fold decrease in MIC [30]. Unfortunately, no reports have been published regarding the antifungal activity of nystatin against postharvest pathogenic fungi.

The objective of this study was to assess the impact of nystatin treatment on the occurrence of gray mold in table grapes that were inoculated with *B. cinerea* conidia. Furthermore, the impact of nystatin on spore development was examined through in vitro cultivation. Additionally, the inhibitory mechanism of nystatin against gray mold in table grapes was investigated through cell viability staining, measuring cell membrane permeability, and a transcriptome analysis. The obtained results could serve as a theoretical foundation for considering nystatin as a viable alternative in the management of gray mold in harvested fruit.

## 2. Materials and Methods

### 2.1. Pathogen Culture

*B. cinerea* (strain B05.10) was kindly provided by Prof. Paul Tudzynski (Molekularbiologie und Biotechnologie der Pilze, Institut für Biologie und Biotechnologie der Pflanzen, Münster, Germany) [31,32]. Potato dextrose agar (PDA) medium (Yuanye Biotechnology, Shanghai, China) was utilized for the cultivation of *B. cinerea* at a temperature of 22 °C under white light (TLD18W/54-765, Philips, Shenzhen, China) [33,34]. After 10 days of cultivation on PDA plates, the fungal spores were extracted by thoroughly soaking the culture surface with sterile distilled water containing 0.05% Tween-20 (Yuanye Biotechnology, Shanghai, China). The spore suspension underwent filtration through a double layer of sterile cheesecloth to effectively remove any adhering mycelia. Subsequently, the concentration of the spores in the suspension was precisely determined using a hemocytometer. Finally, the spore suspension was diluted with potato dextrose broth (PDB) medium (Yuanye Biotechnology, Shanghai, China) to achieve a concentration of 2 × 10^5^ conidia/mL [21].

### 2.2. Fruit

Commercial matured grapes (*Vitis vinifera* L. cv. Victoria), free of mechanical damage, were purchased in 2023 from a fruit store in Xiamen, China.

### 2.3. Reagent

Nystatin (CAS. No. 1400-61-9, 4400 u/mg) was purchased from Yuanye Biotechnology (Shanghai, China).

### 2.4. In Vivo Assessment of Pathogenicity

The table grape fruits were randomly allocated into five groups of 20 grapes each. Before treatment, the grape fruits were soaked in a 2% sodium hypochlorite (Xilong Scientific, Guangzhou, China) solution for two minutes, followed by rinsing with clean water; then, the grapes were carefully transferred to a fume hood for air-drying. During the drying process, the grapes were turned to guarantee uniform drying, all while ensuring there was no impact on their quality. The fruits were wounded at the equator using a sterile nail with a width of 2 mm and a depth of 6 mm. Subsequently, each wound was inoculated with 5 μL of the spore suspension (2 × 10^5^ conidia/mL). After the fruit were thoroughly air-dried, 10 μL of a nystatin solution, with a concentration of 0, 25, 50, 75, or 100 mg/L, was inoculated onto the fruit wounds using a pipettor. Finally, the inoculated fruit were carefully arranged within plastic boxes, subsequently sealed in polyethylene bags, and then stored in a constant-temperature incubator set at 22 °C with the humidity maintained at approximately 95% for 3 d. During this time, the lesion diameters were measured every 24 h post-inoculation (hpi). Each treatment comprised three repetitions.

### 2.5. In Vitro Inhibitory Effect of Nystatin Against B. cinerea

The impact of nystatin on *B. cinerea* mycelial growth and spore germination was evaluated following our previously published methods [21]. The spore suspension (5 μL, 2 × 10^5^ conidia/mL) was spot-inoculated at the center of PDA medium plates (diameter: 9 cm) containing nystatin at different concentrations (0, 0.1, 0.4, 0.8, and 1.2 mg/L), followed by incubation at a temperature of 22 °C. The colony diameter was examined every 24 hpi and the experiment was conducted three times with 5 replicates for each treatment. For the spore germination assay, a spore suspension of *B. cinerea*, containing 1 × 10^6^ conidia/mL, was incubated in PDB medium with varying concentrations of nystatin (0, 0.1, 0.4, 0.8, and 1.2 mg/L) at 22 °C to measure the germination rates and germ tube lengths. A spore was deemed to be germinated when the length of its germ tube exceeded its own diameter. The germination rate was calculated as the percentage of germinated spores out of the total number of spores evaluated. The length of the germ tube was precisely measured using an ocular micrometer [21]. For each field of view, approximately 200 spores were randomly observed, and the entire process was repeated three times to ensure the accuracy and reliability of the results.

### 2.6. Cell Viability Analysis

A conidia suspension of *B. cinerea* (1 × 10^6^ conidia/mL) was cultured in 2 mL of PDB medium with or without nystatin (0, 0.6, 1.2 mg/L) at a controlled temperature of 22 °C. After incubation for 2 h, the spores were stained using 50 mg/L fluorescein diacetate (FDA, Sigma-Aldrich, St. Louis, MO, USA) and subsequently observed under a fluorescence microscope (Leica DM2500 LED, Wetzlar, Germany) [35].

### 2.7. Measurement of Malondialdehyde (MDA) Content, Electrical Conductivity, and Cellular Leakage

MDA, one of the main products of membrane lipid peroxidation, serves as an indicator for the extent of membrane lipid peroxidation. In our study, we employed the thiobarbituric acid (TBA) assay to quantify MDA levels [36], enabling us to assess the impact of nystatin treatment on membrane lipid peroxidation in *B. cinerea*. After incubating the spore suspension for three days on a rotary shaker (Labgic, Beijing, China) set at 22 °C and 200 rpm, different concentrations of nystatin (0, 0.6, and 1.2 mg/L) were added to the spore suspension, followed by further incubation for 0, 2, 4, 6 and 8 h before the measurements were performed.

The electrical conductivity of the spores treated with nystatin was determined using a conductivity meter (EC215, HANNA Instrucment, Shanghai, China) [20]. In brief, 200 mL of the spore suspension (1 × 10^6^ conidia/mL) was cultured in the dark for 3 d on a rotary shaker set at 200 rpm at 22 °C. Following centrifugation (8000× *g*) and washing with sterile water, the mycelia (0.2 g) were resuspended in 15 mL of sterile water containing varying concentrations of nystatin (0, 0.6, and 1.2 mg/L) and then further incubated with shaking (200 rpm) for durations of 0, 2, 4, 6 or 8 h. Subsequently, the electrical conductivity of each culture medium was determined using a conductivity meter. After 20 min of boiling in a water bath, 15 mL of distilled water was added to each group of culture solutions, followed by a second measurement of conductivity. To eliminate any potential confounding influence arising from the background conductivity of nystatin, the results are presented as relative changes compared to the initial measurement. The entire experimental procedure was replicated three times to ensure accuracy and reproducibility.

The leakage of cytoplasmic content from *B. cinerea* after treatment with nystatin was determined following the method from our previous publication [37]. The spore suspension (50 mL) was cultured at 22 °C for 3 d, followed by harvesting. Subsequently, the mycelia were resuspended in 30 mL of sterile water supplemented with nystatin at concentrations of 0, 0.6, and 1.2 mg/L. These suspensions were then cultured at 22 °C for another 0, 2, 4, 6, and 8 h. After centrifugation and removal of the mycelia, the filtrate was used to measure the release of nucleic acids and soluble carbohydrates. The leakage of nucleic acids was quantified using a spectrophotometer by measuring the absorbance at a wavelength of 260 nm. Soluble carbohydrate leakage was determined by utilizing an anthrone reagent as the reference standard, while protein leakage was measured by adhering to the methodology outlined by Zhang et al. (2023) [38].

### 2.8. RNA Sequencing

The details of the inhibitory effect of nystatin on *B. cinerea* was revealed through a comprehensive RNA sequencing analysis. *B cinerea* spores were incubated in 100 mL of PDB (Yuanye Biotechnology, Shanghai, China) for 3 d (200 rpm) and maintained at 22 °C. Subsequently, nystatin was added to achieve a concentration of 1.2 mg/L, and the incubation process was carried out for another 2 h. The mycelia were gathered through centrifugation (8000× *g*) for a duration of 10 min, followed by two washes with PBS buffer (pH 7.4, 10 mM, Sangon Biotech, Shanghai, China) and drying with filter paper, and then they were immediately frozen in liquid nitrogen. The mycelia that were not treated with nystatin (0 mg/L) served as the control. Extraction of total RNA from the collected mycelia was performed using the Trizol reagent. Afterwords, cDNA library construction, transcriptome sequencing, annotation, and analysis were executed in accordance with the protocol outlined by Sun et al., (2021) [39].

### 2.9. RT-qPCR Analysis

Using 1 μg of the aforementioned total RNA, cDNA was synthesized using the PrimeScript RT kit (Takara, Dalian, China). The RT-qPCR was conducted utilizing the StepOne Plus Real-Time PCR system from Applied Biosystems (Carlsbad, CA, USA) and the SYBR premix Ex Taq reagent from Vazyme (Nanjing, China). The RT-qPCR procedures used were as follows: first, the temperature was set to 95 °C for an initial 10 min, followed by 40 cycles, each consisting of 15 s at 95 °C and 30 s at 60 °C. The method of cycle threshold (Ct) 2(−^ΔΔCt^) was applied to determine the gene transcription levels, which allows for quantitative comparisons [40]. The primer pairs for the candidate genes are provided in Table 1.

### 2.10. Statistical Analysis

One-way analysis of variance was conducted using SPSS statistical software 19.0 (Chicago, IL, USA). Data significance was evaluated through either the Student’s *t*-test or Duncan’s multivariate range test. The difference was considered statistically significant when *p* < 0.05.

## 3. Results

### 3.1. Nystatin Effectively Suppressed the Occurrence of Gray Mold on Table Grapes

**Figure 1:**
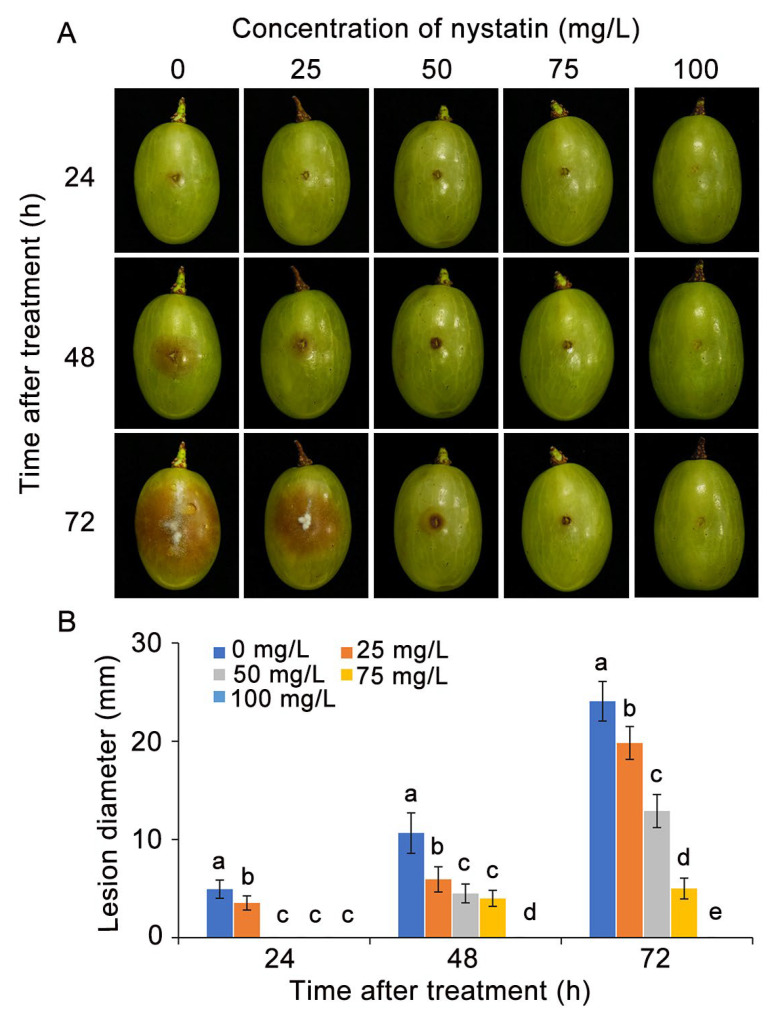
Nystatin dose-dependently reduces the virulence of *B. cinerea* on harvested table grape fruits. (**A**) Lesion development of gray mold on harvested table grape fruits. (**B**) Lesion diameter. The distinct lowercase letters placed above the bars denote a statistically significant variance at *p* < 0.05 (Duncan’s test).

The effect of nystatin on gray mold decay of table grapes was investigated. As shown in Figure 1, after inoculation with *B. cinerea*, the control group exhibited a rapid progression of gray mold decay within 72 h, whereas the nystatin treatment effectively inhibited the progression of gray mold decay in a manner that was dependent on the dosage administered. In contrast to the control group, table grapes treated with 50 mg/L and 75 mg/L nystatin exhibited reductions in lesion diameter of approximately 45% and 75%, respectively, after three days of culture. Moreover, complete inhibition of gray mold rot was achieved at a concentration of 100 mg/L of nystatin.

### 3.2. Nystatin Inhibited the Development of B. cinerea In Vitro

**Figure 2:**
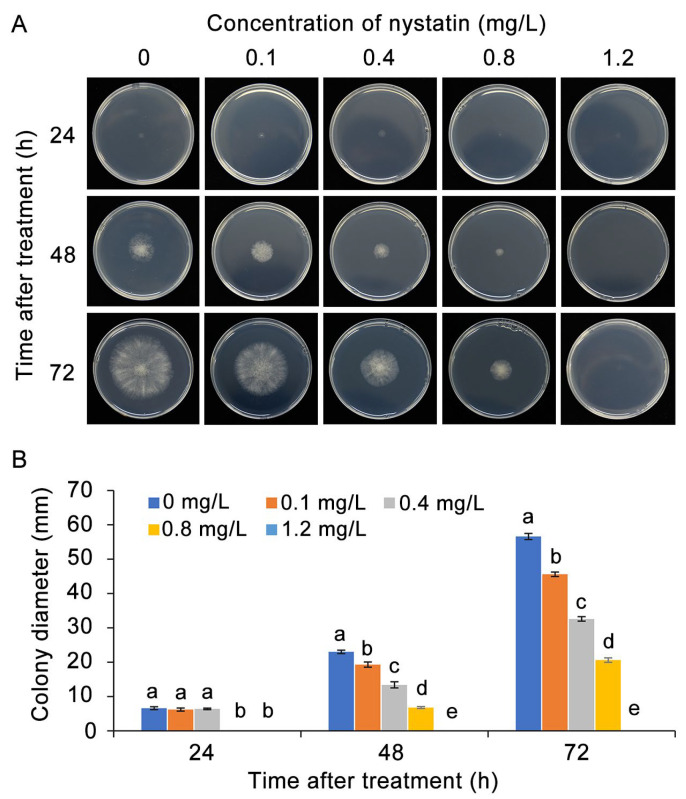
The antifungal activity of nystatin against the vegetative growth of *B. cinerea*. (**A**) Mycelial growth on PDA medium. (**B**) Colony diameter. The distinct lowercase letters placed above the bars denote a statistically significant variance at *p* < 0.05 (Duncan’s test).

The in vitro observations revealed that nystatin exhibited inhibitory properties towards the mycelial growth of *B. cinerea* when cultivated on PDA medium, which displayed a direct correlation with the dosage administered (Figure 2A). In the control culture, the colony diameter gradually expanded during incubation, while the presence of 0.1 mg/L nystatin significantly restricted the mycelial growth of *B. cinerea*. Furthermore, the application of 0.4 mg/L and 0.8 mg/L nystatin resulted in approximately 42% and 64% inhibition rates at 72 hpi, respectively, while 1.2 mg/L nystatin completely suppressed the mycelial growth of *B. cinerea* (Figure 2B).

**Figure 3:**
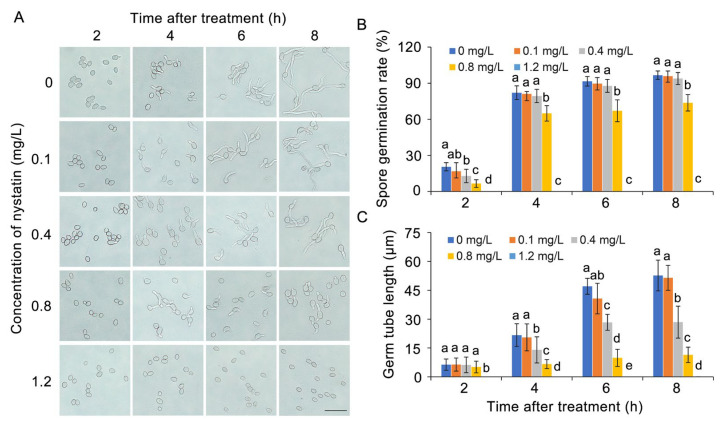
The antifungal activity of nystatin against spore germination of *B. cinerea*. (**A**) Spore morphology following nystatin treatment. Bar = 50 μm. (**B**) Germination rate of *B. cinerea* following treatment with varying concentrations of nystatin. (**C**) Germ tube length of *B. cinerea*. The distinct lowercase letters placed above the bars denote a statistically significant variance at *p* < 0.05 (Duncan’s test).

The results shown in Figure 3A demonstrate that nystatin exhibited an obvious inhibitory effect on the spore germination of *B. cinerea*. Specifically, the spore germination rate was markedly reduced in response to nystatin treatment, showing a direct correlation between the inhibitory effect and the concentration of nystatin. After 8 h of cultivation in PDB medium, spore germination was virtually nonexistent at a concentration of nystatin of 1.2 mg/L (Figure 3B). In addition, the 0.4 mg/L and 0.8 mg/L nystatin treatments reduced the germ tube length of *B. cinerea* by about 46% and 78%, respectively (Figure 3C). Based on the above results, the two concentrations of 0.6 mg/L and 1.2 mg/L were chosen for the subsequent investigations into the inhibitory mechanism of nystatin against *B. cinerea*.

### 3.3. Nystatin Impaired the Cell Viability of B. cinerea

**Figure 4:**
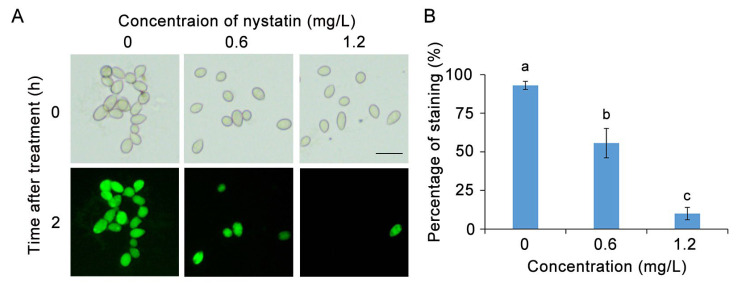
Nystatin decreases the cell viability of *B. cinerea*. (**A**) Microscopic observations of FDA staining of *B. cinerea* (bar = 20 μm). (**B**) Percentages of stained *B. cinerea*. The distinct lowercase letters placed above the bars denote a statistically significant variance at *p* < 0.05 (Duncan’s test).

The cell viability of spores treated with nystatin was assessed using FDA staining. Under a fluorescence microscope, viable cells are stained green by the FDA. The majority of *B. cinerea* cells in the control group exhibited a high fluorescence intensity upon FDA staining, whereas the cells treated with nystatin displayed minimal staining (56% for 0.6 mg/L nystatin treatment and 10% for 1.2 mg/L nystatin treatment) (Figure 4A,B). The results suggested that a majority of the spores of *B. cinerea* experienced a decrease in cellular viability.

### 3.4. Nystatin Treatment Had an Impact on the Production of MDA, Electrical Conductivity, and Cellular Leakage in B. cinerea

**Figure 5:**
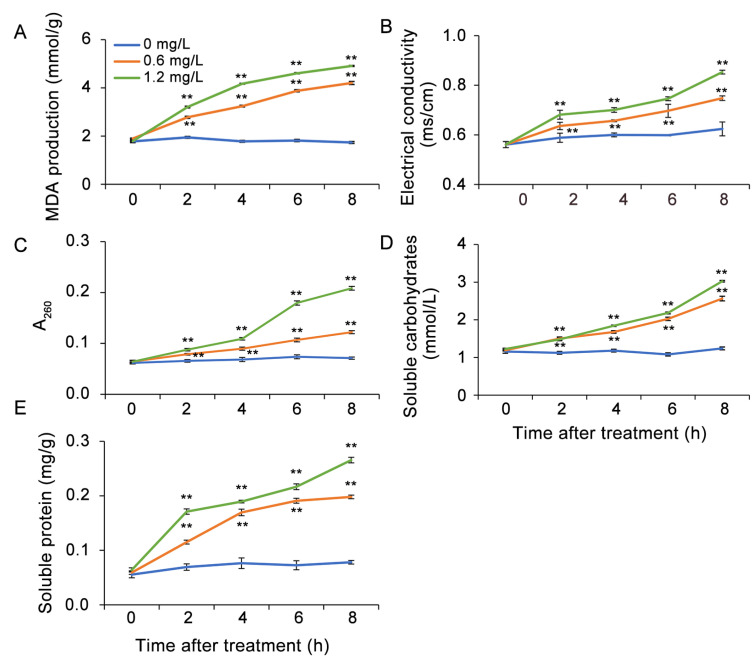
Nystatin treatment causes membrane lipid peroxidation and cellular leakage of *B. cinerea*. (**A**) MDA production. (**B**) Electrical conductivity. (**C**) Nucleic acid content. (**D**) Soluble carbohydrate content. (**E**) Soluble protein content. The asterisks indicate statistically significant divergences from the control group, as ascertained by the Student’s *t*-test (** *p* < 0.01).

To assess the peroxidation of membrane lipids in *B. cinerea* following nystatin treatment, the MDA content was quantified. The production of MDA showed an increasing trend during the 8 h incubation with nystatin and exhibited a positive correlation with the concentration of nystatin. Conversely, the control group exhibited sustained low levels of MDA at approximately 0.36 μmol/g (Figure 5A). These findings indicated that treatment with nystatin induced the peroxidation of membrane lipids in *B. cinerea*.

Electrical conductivity is widely recognized as a crucial indicator for assessing the permeability of the plasma membrane. In the present investigation, we examined the influence of nystatin on the electrical conductivity of *B. cinerea* (Figure 5B). During the entire experimental period, the control group consistently maintained a low level of electrical conductivity for *B. cinerea*. However, a rise in the concentration of nystatin led to a corresponding elevation in the electrical conductivity of *B. cinerea*, indicating an impairment in membrane permeability.

Given the enhanced MDA content and electrical conductivity of nystatin-treated conidia, we further investigated the cellular leakage of nucleic acids, soluble carbohydrates, and soluble proteins from *B. cinerea*. Similarly, the application of nystatin resulted in cytoplasmic leakage, with the release rate gradually increasing throughout the entire experiment. Furthermore, there was a positive correlation between the dose of nystatin and the release of nucleic acids, soluble carbohydrates, and soluble proteins (Figure 5C–E).

### 3.5. Transcriptome Analysis of B. cinerea Following Nystatin Treatment

RNA-seq analysis was used to investigate the underlying inhibitory mechanisms of nystatin at a concentration of 1.2 mg/L. The average total number of clean bases per sample was 12.4 G, with approximately 98.79% of the clean reads successfully mapped to the reference genome of *B. cinerea* for gene expression analysis. The comparison between the control and nystatin treatment groups revealed a total of 4351 differentially expressed genes (DEGs), among which, 2149 were up-regulated and 1923 were down-regulated (Figure S1, Table S1).

**Figure 6:**
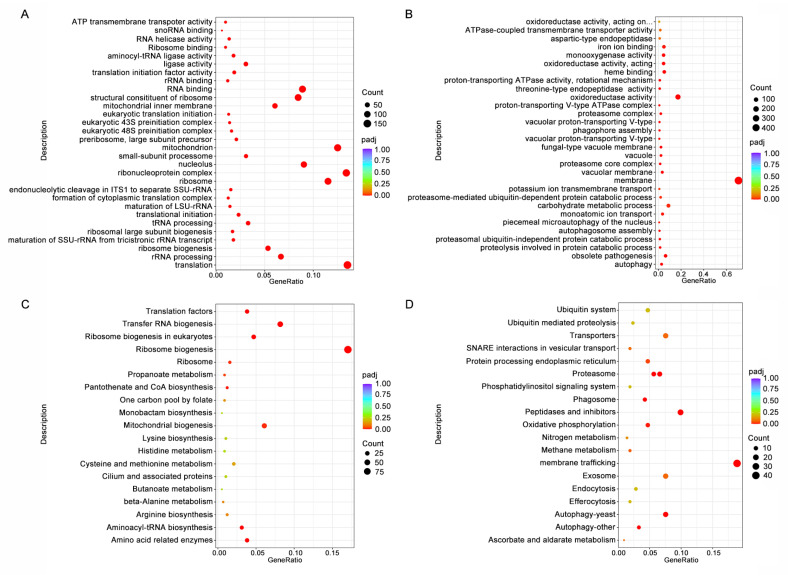
Transcriptome analysis of differentially expressed genes (DEGs) in *B. cinerea* under 1.2 mg/L nystatin treatment compared to the control. GO classification analysis results for the up-regulated DEGs (**A**) and down-regulated DEGs (**B**). KEGG pathway analysis results for the up-regulated DEGs (**C**) and down-regulated DEGs (**D**). The size of the dots corresponds to the number of enriched genes, while the color intensity reflects their statistical significance.

The GO enrichment analysis of the DEGs revealed that the majority of the up-regulated DEGs are associated with oxidoreductase activity, nucleotide binding, RNA binding, and catalytic activity. Furthermore, a considerable number of up-regulated DEGs are annotated as components of the nucleolus, ribosomes, and mitochondria (Figure 6A). Among the 1923 down-regulated DEGs, 496 are annotated as membrane components. Additionally, there were 166 DEGs that possess oxidoreductase activity, 100 DEGs involved in transmembrane transport, and 63 DEGs associated with the carbohydrate metabolic process (Figure 6B).

Based on the KEGG enrichment analysis of the up-regulated DEGs, a high proportion of DEGs are involved in ribosome biogenesis (98 genes), transfer RNA biogenesis (47 genes), and mitochondria biogenesis (35 genes). In addition, genes related to amino acid biosynthesis and metabolism were remarkably up-regulated (Figure 6C). The most prominent pathway for the down-regulated DEGs was membrane trafficking. Moreover, treatment with nystatin also lead to a down-regulation in the expression of genes involved in the activity of peptidases, transporters, and inhibitors; autophagy; exosome biogenesis; and oxidative phosphorylation (Figure 6D).

**Figure 7:**
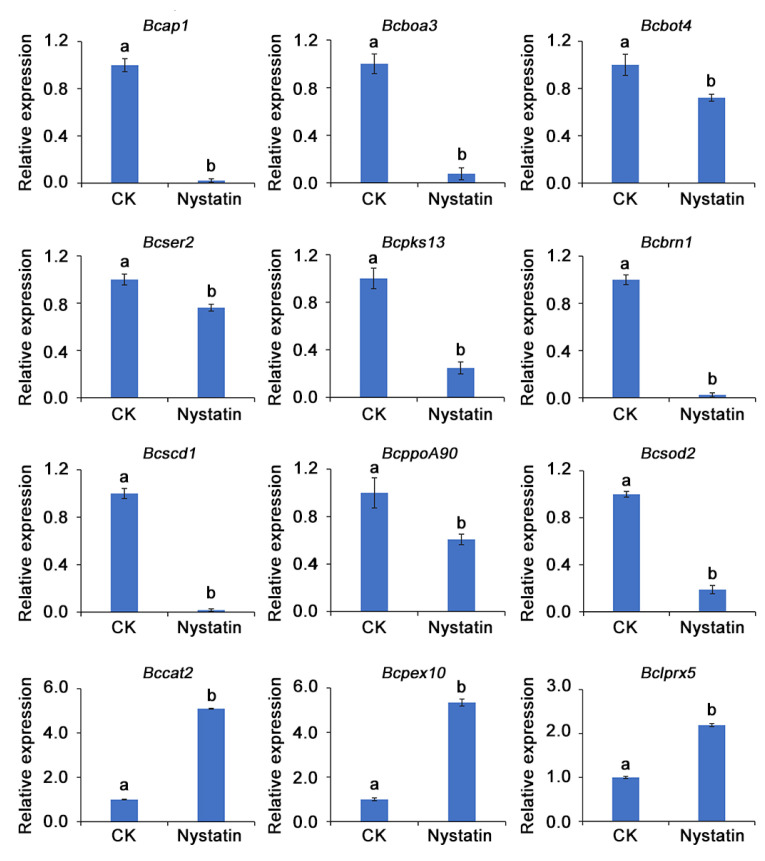
The relative expression levels of twelve DEGs related to secreted proteolytic activity, phytotoxin biosynthesis, melanin biosynthesis, and oxidoreductase activity were validated by RT-qPCR. The distinct lowercase letters placed above the bars denote a statistically significant variance at *p* < 0.05 (Duncan’s test).

The transcriptional levels of twelve genes related to proteolysis, pathogenesis, phytotoxin biosynthesis, melanin biosynthesis, oxidoreductase activity, and melanin metabolic process were further validated using RT-qPCR analysis. As depicted in Figure 7, following nystatin treatment, the expression levels of *Bcap1*, *Bcboa3*, *Bcbot4*, *Bcser2*, *Bcpks13*, *Bcbrn1*, *Bcscd1*, *BcppoA90*, and *Bcsod2* were down-regulated while those of *Bccat2*, *Bcpex10*, and *Bcprx5* were up-regulated. The observed results were consistent with the RNA-seq data, confirming the credibility of the transcriptome analysis.

### 3.6. Application of Nystatin Down-Regulated the Expression of Genes Associated with Autophagy

**Figure 8:**
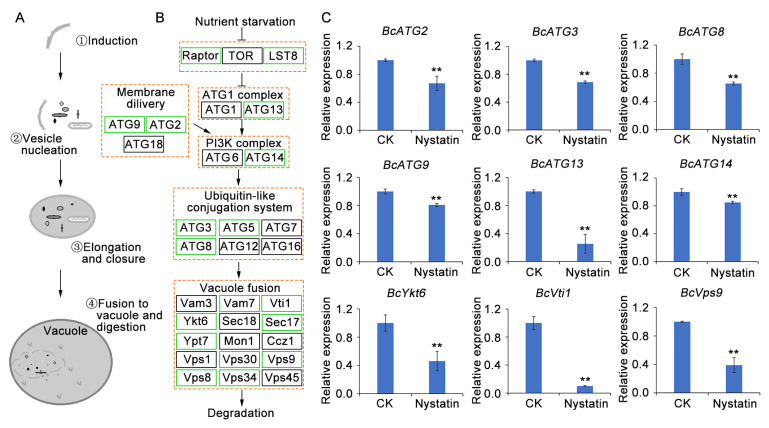
Nystatin disrupts the autophagic process. (**A**) Schematic depiction of autophagy. (**B**) The DEGs involved in the autophagy pathway, where green boxes indicate down-regulated genes and black boxes represent stable genes. Table S1 provides detailed information on these DEGs. (**C**) RT-qPCR analysis of 9 autophagy-related genes. The asterisks indicate statistically significant divergences from the control group, as ascertained by the Student’s *t*-test (** *p* < 0.01).

Autophagy is an evolutionarily conserved degradation pathway that involves the degradation of cytoplasmic organelles, long-lived proteins, and other macromolecules, as well as nutrient recycling [41,42]. Autophagy initiation, autophagosome formation, and autophagosome–vacuole fusion are orchestrated by the sequential regulation of autophagy-related genes, as depicted in Figure 8A,B. In this study, GO enrichment of the DEGs from the transcriptome analysis revealed that a total of 20 autophagy-related genes and 7 genes involved in autophagy assembly were down-regulated (Table S1). Subsequently, RT-qPCR was employed to validate the expression profiles of nine differentially expressed autophagy-associated genes identified in the transcriptome analysis. The findings revealed a remarkable down-regulation of *BcATG2*, *BcATG3*, *BcATG8*, *BcATG9*, *BcATG14*, *BcATG14*, *BcYkt6*, *BcVti1*, and *BcVps9* subsequent to nystatin treatment, which aligned with the transcriptome data (Figure 8C), indicating that the nystatin treatment disrupted the autophagy process.

## 4. Discussion

*B. cinerea*-induced gray mold represents the most economically significant postharvest disease that affects table grapes [43]. This is primarily due to the contamination of the fruit surface by *B. cinerea* spores, which severely impacts both the quality and shelf life of table grapes, resulting in substantial economic losses [33,44]. A dose-dependent suppressive effect of nystatin against *B. cinerea* was observed under in vitro conditions in the present study. Furthermore, nystatin exhibited remarkable efficacy in managing postharvest gray mold in table grapes. Previous studies have demonstrated that nystatin, a polyene antifungal agent, has an amphipathic structure that binds to ergosterol, effectively transforming the fluid cellular membrane of fungal cells into a more crystalline form, thus enhancing membrane permeability [45,46]. In order to understand how nystatin inhibits the virulence of *B. cinerea*, cell viability, membrane lipid peroxidation, and membrane permeability were measured. As a result, the viability of cells was significantly decreased upon nystatin treatment, accompanied by an elevation in membrane lipid peroxidation and enhanced membrane permeability, thereby indicating a pronounced impact of nystatin on cell function. The subsequent experiments demonstrated the release of cellular contents, including nucleic acids, soluble carbohydrates, and soluble proteins, from *B. cinerea* cells, providing further evidence for the disruption of the plasma membrane. Moreover, the transcriptome data revealed notable changes in the expression patterns of numerous genes related to the structure and function of the membrane. These results imply that the plasma membrane of *B. cinerea* may serve as the primary target for nystatin.

The ribosomes are intracellular manufacturing units that play a crucial role in synthesizing proteins that are vital for cellular growth and survival. Moreover, mitochondria serve as the hub for integrating multiple metabolic functions, including lipid metabolism, iron homeostasis, energy production, and cell wall biosynthesis, all of which are closely related to fungal virulence [47,48]. The transcriptome data showed that the presence of nystatin had an impact on the composition and biogenesis of ribosomes and mitochondria, which is in agreement with the observed suppression of vegetative growth and virulence of *B. cinerea* under nystatin treatment. Furthermore, mitochondria are regarded as the primary origin and target of intracellular reactive oxygen species (ROS) [49]. The primary role of ROS, as crucial second messengers, is to regulate the cellular redox state and downstream signal transduction, which is indispensable for pathogen growth, development, and infection [50]. Increasing evidence suggests that various exogenous stresses can induce oxidative damage in fungal pathogens, leading to a decline in their pathogenicity [51,52,53]. The transcriptome analysis showed that 166 genes related to oxidoreductase activity were down-regulated, while 188 were up-regulated, indicating that the redox homeostasis in *B. cinerea* was disrupted following nystatin treatment, leading to a reduction in the virulence of *B. cinerea*.

In bacteria, viruses, and fungi, peptidases constitute approximately 1–5% of the genome [54]. During the infection process, fungi possess the capability to secrete a diverse range of extracellular peptidases, which play a pivotal role as virulence factors [8,55]. The KEGG enrichment analysis conducted in this study revealed that a total of 21 genes related to the activity of peptidases and inhibitors were down-regulated in the nystatin-treated group, suggesting that nystatin exerted an impact on the secretion of peptidases in *B. cinerea*, thereby influencing its pathogenicity. Furthermore, proteasomes occupy a pivotal position in executing the majority of cytoplasmic and nuclear proteolysis processes in eukaryotes [56]. Eukaryotic cells employ two primary protein degradation pathways: the ubiquitin–proteasome system (UPS) and the lysosomal proteolysis [57]. The ubiquitin–proteasome pathway is responsible for the degradation of most intracellular proteins and exhibits high specificity [58]. The ubiquitin genes in fungal pathogens are essential for fungal development, stress resistance, and virulence. For instance, Fbp1, as an integral component of the SCF E3 ligase complex, is indispensable for the full virulence of *Cryptococcus neoformans* which causes fungal meningitis in humans. Additionally, Fbp1 is also essential for the invasive growth and virulence of the plant fungal pathogen *Fusarium oxysporum* [59,60,61]. Recently, *Candida albicans*, a fungal pathogen capable of undergoing morphological transitions to adapt to diverse environmental stimuli, has been found to possess two ubiquitin genes [62]. *UBI3*, a hybrid ubiquitin fusion protein-encoding gene, is vital for the growth of *C. albicans*. *UBI4* deficiency induces a morphological transition from yeast to mycelium, a transformation that significantly contributes to its pathogenicity [63,64]. In the current study, it was noted that the nystatin treatment resulted in a down-regulation in the transcription of genes associated with the proteasome, ubiquitin system, and ubiquitin-mediated proteolysis in *B. cinerea*, suggesting an abnormal UPS system for protein degradation, ultimately reducing its pathogenicity.

The process of autophagy involves the vacuole/lysosome-mediated degradation pathway, which is highly conserved and facilitates the removal and recycling of various cellular components, such as cytoplasm, macromolecules, and dysfunctional organelles [65,66]. Extensive studies have illuminated the vital functions of autophagy. On the one hand, it acts as a key regulator in preserving cellular homeostasis, and on the other, it plays an indispensable role in governing the growth, morphology, development, and pathogenicity of filamentous fungi [41,42,67]. Furthermore, the crucial role of autophagy-associated genes, namely *BcATG1*, *BcATG2*, *BcATG4*, and *BcATG8*, in facilitating the vegetative differentiation and pathogenicity of *B. cinerea* has been confirmed [68,69,70,71]. Additionally, a previous study has shown that spm1, a subtilisin-like serine protease, is localized within vacuoles and is required for infection-related autophagy in *Magnaporthe oryzae*. Knockout of *spm1* leads to appressorium formation and infectious growth defects [72]. Moreover, a Bcser2 (a subtilisin-like protease) defect significantly attenuated the development and virulence of *B. cinerea* [73]. These all suggest that autophagy can serve as a novel target for the development of antifungal compounds. In recent years, numerous studies have demonstrated the efficacy of antifungal agents, such as plumbagin [74], 3-octanol [38], 2,3-butanedione [33], and rapamycin [20], in controlling gray mold through modulating the autophagic process of *B. cinerea*. The findings of the present study demonstrate that nystatin treatment significantly down-regulates the expression of autophagy-associated genes in fungal cells, as evidenced by the results of the transcriptome analysis and RT-qPCR assay. Interestingly, the antifungal agents reported in the past all activated the autophagy process of *B. cinerea*, whereas the nystatin treatment exhibited the opposite effect. These results strongly suggest that the reduced virulence observed in *B. cinerea* may be due to the interference of nystatin with the autophagy process.

## 5. Conclusions

Microbially derived agricultural antibiotics, renowned for their high efficacy and eco-friendliness, serve as a cornerstone in the realm of biological pesticides. Thus, the diligent screening and development of novel microbially derived agricultural antibiotics hold paramount importance in advancing the evolution of biological pesticides and fostering sustainable agricultural production. In the present study, nystatin was found to exhibit dose-dependent efficacy in controlling the mycelial growth and spore germination of *B. cinerea*, as well as its pathogenicity on table grapes. Primarily, the antifungal activity was attributed to the disruption of cell membrane permeability, resulting in cytosolic content leakage and eventually causing cell death. Moreover, nystatin disturbed the expression of genes related to ribosome and mitochondria composition and biogenesis, the UPS system that functions in protein degradation, as well as autophagy activity, ultimately diminishing the pathogenicity of *B. cinerea*. This study’s findings suggest that nystatin is a viable postharvest treatment for fresh produce, and provides a theoretical basis for its application.

## Figures and Tables

**Table 1:** Primers used in this study.

Gene Name	Primer F (5′–3′)	Primer R (5′–3′)
*Bcap1*	GAGCCCACTAAACCTGGACC	CTGGGACTTCACCGTTCTCC
*Bcboa3*	TTCAGCTCGGCTCAGTCTTG	TTTGCGGATCTGGCTCTGTT
*Bcbot4*	GCTCGTCACCAAGAACCAGA	CATGAGCCGTCATCTCCTCC
*Bcser2*	GTCATCGAAGAGGTCCGCAA	TCTGTGGGAGATACGAGCCA
*Bcpks13*	GCCAAAAACCGTTGCCATCT	CAGTCCATGGAGTTGGGCAT
*Bcbrn1*	AGGATGTCACCGAGGAGGAA	ACTCCCTTAGCCTGACCAGT
*Bcscd1*	CCCAACATCTTCTCGGTGCT	GGCATGACTGTGACCCTTCA
*BcppoA90*	TATCTCCGCACAATCGTCGG	CACATTGGTTCCCGACTCCA
*Bcsod2*	ACCTTGTTGGACGGTGTTGT	CAAGCTGCTCTGGACACTGA
*Bccat2*	AAGGCAAACCCATCAAACGC	ACCGTGCCAGTATTGATGGG
*Bcpex10*	CCTGGAAGTTCCGCAGTTCT	GGAATGGGGAATAGCCGAGG
*Bcprx5*	GGCATTTGCTGAACCCATGG	CGGGTATCTCGTCAGCACTC
*BcATG2*	AGTGGATGTGCAGTTGAGGG	TCTAGCCCCTGGTTCTTCGA
*BcATG3*	ACGAAGATGGTTGGCTGAGG	CATCGTCTTCTCTCTCGCCC
*BcATG8*	ACGAGCACCCATTCGAGAAG	TGTCGATAGTGGCGATGTCG
*BcATG9*	GCGCTTCCAAAGAAAGCACA	TGGCTGCGTCATCTTGATGT
*BcATG13*	ACCACGAAGACCAAGTCGAC	ACTTGACCTTCGAGCGTGAG
*BcATG14*	GTAGACCTGAGGTTGATGAACAC	ATCTCTGCAGGCAACCGAACAG
*BcYkt6*	AATATCCAGCCCTCGTTGCC	TGAGGGTTGGGGTTGAATCG
*BcVti1*	AGACCGAAGCTCCCAGAGAT	TTGGTAGCCATCCTACGGGA
*BcVps19*	AGTAATCGAAGCGGCGGTAG	GGTTGAGTGAGTTGCCGAGA

## Data Availability

The original contributions presented in the study are included in the article/Supplementary Materials, further inquiries can be directed to the corresponding author.

## Supplementary Materials

The following supporting information can be downloaded at: https://www.mdpi.com/article/10.3390/foods13223624/s1, Figure S1. Transcriptome analysis of DEGs following nystatin treatment. Table S1. Differential gene expression between control and nystatin treatment group.
