# Supplementary material for: Efficacy and Molecular Mechanisms of Nystatin Against Botrytis cinerea on Postharvest Table Grape
Source: Foods. 2024 Nov 13;13(22):3624. doi: 10.3390/foods13223624 (PMC11593271; doi:10.3390/foods13223624)
Supplement: Supplementary file 1 [file foods-13-03624-s001.zip › Supplementary Figures.pdf]

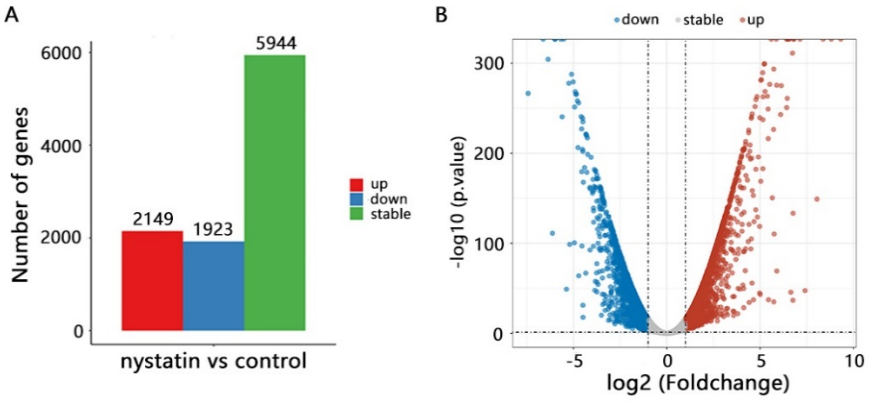

Figure S1. Transcriptome analysis of DEGs following nystatin treatment. (A) Numbers of DEGs in control and nystatin-treated group. (B) Volcano map of DEGs. The red and blue colors correspond to up-regulated and down-regulated genes, respectively.
